# Polishing Performance and Removal Mechanism of Core-Shell Structured Diamond/SiO_2_ Abrasives on Sapphire Wafer

**DOI:** 10.3390/mi13122160

**Published:** 2022-12-07

**Authors:** Guangen Zhao, Yongchao Xu, Qianting Wang, Jun Liu, Youji Zhan, Bingsan Chen

**Affiliations:** 1 School of Materials Science and Engineering, Fujian University of Technology, Fuzhou 350118, China; 2 Fujian Key Laboratory of Intelligent Machining Technology and Equipment, Fujian University of Technology, Fuzhou 350118, China

**Keywords:** polishing, sapphire, core shell, composite abrasives, surface roughness, material removal rate

## Abstract

Corrosive and toxic solutions are normally employed to polish sapphire wafers, which easily cause environmental pollution. Applying green polishing techniques to obtain an ultrasmooth sapphire surface that is scratch-free and has low damage at high polishing efficiency is a great challenge. In this paper, novel diamond/SiO_2_ composite abrasives were successfully synthesized by a simplified sol-gel strategy. The prepared composite abrasives were used in the semi-fixed polishing technology of sapphire wafers, where the polishing slurry contains only deionized water and no other chemicals during the whole polishing process, effectively avoiding environmental pollution. The experimental results showed that diamond/SiO_2_ composite abrasives exhibited excellent polishing performance, along with a 27.2% decrease in surface roughness, and the material removal rate was increased by more than 8.8% compared with pure diamond. Furthermore, through characterizations of polished sapphire surfaces and wear debris, the chemical action mechanism of composite abrasives was investigated, which confirmed the solid-state reaction between the SiO_2_ shell and the sapphire surface. Finally, applying the elastic-plastic contact model revealed that the reduction of indentation depth and the synergistic effect of chemical corrosion and mechanical removal are the keys to improving polishing performance.

## 1. Introduction

Sapphire, composed of single crystal alumina oxide (α-Al_2_O_3_), is an ideal material for infrared windows and aerospace [1,2] and is the main substrate material for optoelectronic devices, large-scale integrated circuits [3,4,5,6], and superconducting films due to its excellent mechanical and optical properties, such as high hardness, strong light transmittance, and stable chemical inertness. In particular, as the substrate material of GaN-based light-emitting diodes (LEDs), sapphire wafers have strict requirements for processing accuracy and surface quality, including nanoscale surface roughness, damage-free, and scratch-free [7]. However, given the high hardness and chemical inertia of sapphire [8,9], it’s a great challenge to achieve satisfactory processing results.

The widely used free abrasive polishing is a traditional material removal strategy, which can provide a smooth surface in the field of electronic device substrate manufacturing [10]. However, because of the high hardness and brittleness of sapphire, the free abrasive process has the disadvantages of uncontrollable trajectory [11], low removal efficiency, and easy agglomeration, which will undoubtedly affect the polishing effect, resulting in high roughness and heavy damage on sapphire surfaces [12]. In addition, the free abrasive polishing of sapphire wafers usually applies strong acids, alkalis and toxic chemicals, leading to environmental pollution [13]. Among the reported nontraditional polishing technologies, the semi-fixed polishing pad using diamond abrasive has attracted great attention, which effectively avoids the problems of uncontrollable trajectory and the agglomeration of free abrasive [14]. The abrasives in the semi-fixed pad exhibited “yielding effects”, for which surface damage and scratches induced by the larger abrasives can be reduced or even eliminated. Furthermore, in the whole polishing process, the slurry contains only deionized water, without any other chemicals, effectively avoiding environmental pollution. Hence, a semi-fixed abrasive polishing pad is one of the most promising polishing tools for processing hard and brittle materials such as sapphire, SiC, GaN, etc. [15]. Nevertheless, the relatively soft polishing pad inevitably reduces the polishing efficiency of the inner abrasive, making it difficult to achieve perfect surface quality with a high material removal rate (MRR) even when using hard abrasives. In order to overcome these problems, scholars have conducted in-depth research on polishing abrasives and made lots of significant progress.

Recent advances in composite abrasives with core-shell structures have provided a new direction for obtaining a supersmooth surface and a high MRR [16,17,18,19]. The core-shell composite abrasives overcome the limitations of single hard abrasives with many deep scratches and single soft abrasives with a low material removal rate, and they give full play to the excellent characteristics of different abrasives. Lu et al. [20] developed novel diamond/akageneite composite abrasives with a core-shell structure, which possessed stronger adhesion to the semi-fixed polishing pad, and the surface quality of sapphire has also been improved. Specifically, the surface roughness (Ra) of sapphire polished by diamond/akageneite composite abrasives was reduced from 1.70 nm to 1.39 nm, which is about 12.6% lower than that of pure diamond. The MRR of the diamond/akageneite composite abrasives is similar to that of diamond, which is about 0.28 nm/min. In our previous work [21], the prepared Al_2_O_3_/SiO_2_ composite abrasives achieved excellent polishing performance, along with a 20.2% decrease in surface roughness, and the MRR was increased by more than 5.1% compared with pure Al_2_O_3_. The improvement in the MRR may be attributed to the solid-state reaction of the SiO_2_ shell with the sapphire surface during polishing, resulting in a softened layer that can be easily removed by the mechanical action of the hard core. With a Mohs hardness of 10, diamond has a higher removal effect than Al_2_O_3_ (Mohs hardness of 9), which means that using diamond as the core material is expected to further improve MRR. However, the polishing performance of diamond/SiO_2_ composite abrasives on the sapphire wafer has not been reported. In addition, research on the polishing behavior and material removal mechanism of core-shell composite abrasives is still insufficient.

In this study, the diamond/SiO_2_ composite abrasives were successfully synthesized via a simplified sol-gel method and then characterized by field emission scanning electron microscopy (FESEM), transmission electron microscope (TEM) and EDS energy spectrum, X-ray diffraction (XRD), and Fourier transform infrared spectra (FT-IR), respectively. Subsequently, the polishing performance of pure diamond and diamond/SiO_2_ composite abrasives on sapphire wafers was explored by using semi-fixed abrasive polishing pad under the same polishing parameters. The polishing results were investigated from the aspects of surface morphology, surface roughness, the material removal rate (MRR), and residual stress. Finally, combined with TEM and X-ray photoelectron spectroscopy (XPS), the polishing behavior and material removal mechanism of composite abrasives on sapphire wafers were discussed in terms of mechanical action and chemical corrosion.

## 2. Materials and Experimental Methods

### 2.1. Chemicals and Materials

Commercial diamond particles as the core material of composite abrasives, with a nominal particle size of 3 μm, were supplied by Yvxing Micro diamond Co. Ltd. (Zhengzhou, China). Tetraethyl orthosilicate (TEOS, AR), offered by Shanghai Yien Chemical Technology Co., Ltd. (Shanghai, China), was used as raw material to provide silicon shell through hydrolysis polycondensation and other reactions. Other chemicals, including ammonia solution (NH_3_·H_2_O, 25–28%) and absolute ethanol (C_2_H_5_OH, AR), were purchased from Shanghai Chemical Reagent Co., Ltd. (Shanghai, China).

### 2.2. Synthesis of Diamond/SiO_2_ Composite Abrasives

Diamond/SiO_2_ core-shell composite abrasives were synthesized via a facile sol-gel strategy on the basis of the hydrolysis and polycondensation reaction of TEOS. Firstly, a certain amount of diamond particles was added to the beaker containing absolute ethanol and was dispersed under ultrasound for 20 min until a uniform suspension was formed. Afterward, the diamond suspension was transferred to a thermostatic water bath; under continuous stirring, ammonia solution and deionized water were slowly dropped into the diamond suspension. After magnetic stirring at 30 °C for 15 min, TEOS was drop by drop added into the above mixed solution. The reaction was carried out at 30 °C for 12 h, during which magnetic stirring at low speed was maintained. Subsequently, the resultant precipitates were collected by centrifugation and washed three times with deionized water and anhydrous ethanol, separately. Finally, they were dried at 60 °C for 12 h, with which diamond/SiO_2_ core-shell composite abrasives were obtained.

By means of a simplified sol-gel method, tetraethyl orthosilicate (TEOS, Si (OC_2_H_5_)_4_) was catalyzed by ammonia to form a SiO_2_ shell on the surface of diamond particles through hydrolysis and polycondensation. The formation of the SiO_2_ shell can be summarized by the following chemical equations [22]:




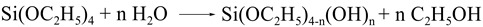

(1)






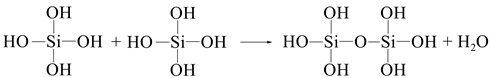

(2)






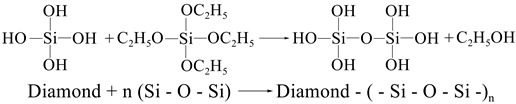

(3)



Combined with the above chemical formulas, TEOS hydrolyzes to generate silanol, which can also be regarded as the release of active monomers, leading to a nucleation phenomenon [23]. On account of the existence of multiple O-H functional groups on the surface of diamond particles, coupled with its high specific surface area and high specific surface energy, the O-H functional groups are preferentially combined with silanol groups through a condensation reaction, which is the key for SiO_2_ to nucleate on the surface of diamond. Subsequently, as-nucleated SiO_2_ particles slowly gathered on the surface of diamond particles to form a discontinuous coating. Given the colloidal stability, there is a balance between the hydrolysis of TEOS and the primary particles. In other words, with the progress of reaction, the active monomer increases continuously, and the SiO_2_ seeds on the diamond surface grow further to form a uniform and dense SiO_2_ shell.

### 2.3. Characterizations

FESEM (NovaNanoSEM450, FEI Ltd., Natural Bridge Station, VA, USA) was employed to observe the external morphology of pure diamond and composite abrasives. The microstructure and elemental composition of abrasives were characterized by using the TEM (JEM-2100, JEOL Ltd., Tokyo, Japan) equipped with EDS (Oxford X-MaxN, Oxford Instruments Ltd., Abingdon, UK). XRD (D8ADVANCE, Bruker Ltd., Ettlingen, Germany) was adopted to analyze the phase components of pristine and composited abrasives by using Cu Kα radiation. To interpret the surface functional groups of abrasives, the spectra were measured within the wavenumber range of 400 cm^−1^ to 4000 cm^−1^ on FT-IR (Nicolet 6700, Thermo Ltd., Waltham, MA, USA).

### 2.4. Polishing Tests

**Figure 1:**
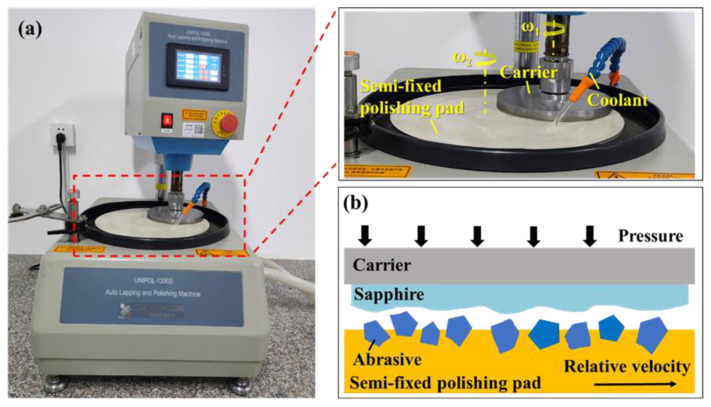
(**a**) Device diagram and (**b**) schematic diagram of the polishing process.

Commercially available single crystal sapphire wafers (C-M plane; two-inch in diameter; Mohs hardness of 9) with original surface roughness values (Ra) of 10 ± 1 nm were bought from Wuxi Jingdian Semiconductor Material Co., Ltd. (Wuxi, China). Polishing texts of sapphire wafers were conducted on a rotary-type polishing tester (UNIPOL-1200S, Shenyang Kejing Co., Ltd., Shenyang, China) with a semi-fixed abrasive polishing pad. The abrasives (5 wt%) were evenly dispersed in a flexible matrix with unsaturated resin as the main component through full mixing and stirring, and then the semi-fixed polishing pad with a diameter of 300 mm was prepared through processes such as screeding and curing. Pure diamond and diamond/SiO_2_ core-shell composite abrasives were separately used as abrasives for the semi-fixed flexible polishing pad. Before polishing, sapphire wafers were several times ultrasonically cleaned in deionized water and absolute ethanol to remove natural oxides and pollutants from their surfaces. The polishing parameters were described as follows: the polishing pressure was set as 5 kg, the polishing time was 3 h, and the rotation speed of the workpiece and that of the polishing pad were 60 rpm and 120 rpm, respectively. No chemicals were used in the polishing process, and only deionized water was used as coolant. After polishing, the sapphire wafers were cleaned repeatedly with deionized water and ethanol under sonication, and then they were dried in a drying oven. The schematics of the polishing process are shown in Figure 1.

The atomic force microscope (AFM, Dimension Icon, Bruker Ltd., Germany) was utilized to investigate the surface topography and profile curve of the sapphire wafers before and after polishing, with an accuracy of 0.01 nm. During the polishing process, the contact surface roughness meter (MarSurf GD25, Mahr Ltd., Germany), with 0.1 nm accuracy, was used to measure the surface roughness (*Ra*) of workpieces every half an hour. For each machined workpiece, surface roughness (*Ra*) is the average value of 10 areas evenly distributed on the sapphire wafer surface. 

To further analyze the material removal mechanism of sapphire wafers, the surface elements and existing forms of polished sapphire wafers were characterized by XPS (ESCALAB 250XI, Thermo Ltd., USA). TEM was used to analyze the wear debris removed from the surface of sapphire wafers during polishing. The material removal rate (nm/min) was calculated by Equation (4), and the masses (the average value of three measurements) of sapphire wafers before and after polishing were tested by an electron balance with 0.01 mg precision (GE0505, Shanghai YoKe, Ltd., Shanghai, China).

(4) $$MRR=\frac{10^{7}\times \Delta m}{\rho \times 2.54^{2}\times \pi \times t}$$

Here, Δm (mg) is the mass loss of sapphire wafers before and after polishing, ρ (3.98 g/cm^3^) is the sapphire density, and *t* (min) is the polishing time.

## 3. Results and Discussion

### 3.1. Characterizations of the Diamond/SiO_2_ Composite Abrasives

**Figure 2:**
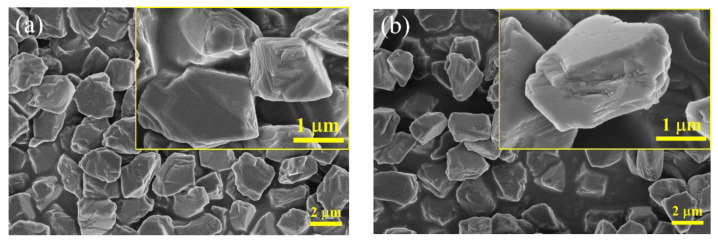
FESEM images of (**a**) pure diamond and (**b**) diamond/SiO_2_ abrasive particles.

FESEM images of pure diamond and diamond/SiO_2_ composite abrasives are illustrated in Figure 2. The morphology of pure diamond shown in Figure 2a demonstrates that the pristine diamond particles have a uniform shape and smooth surface. As can be seen in Figure 2b, the prepared diamond/SiO_2_ composite abrasives have good dispersion and no agglomeration. It is also noticed that there are no SiO_2_ microspheres nucleated separately in Figure 2b, suggesting that the deposition of SiO_2_ onto the diamond occurred in the form of a SiO_2_ network rather than SiO_2_ particles.

**Figure 3:**
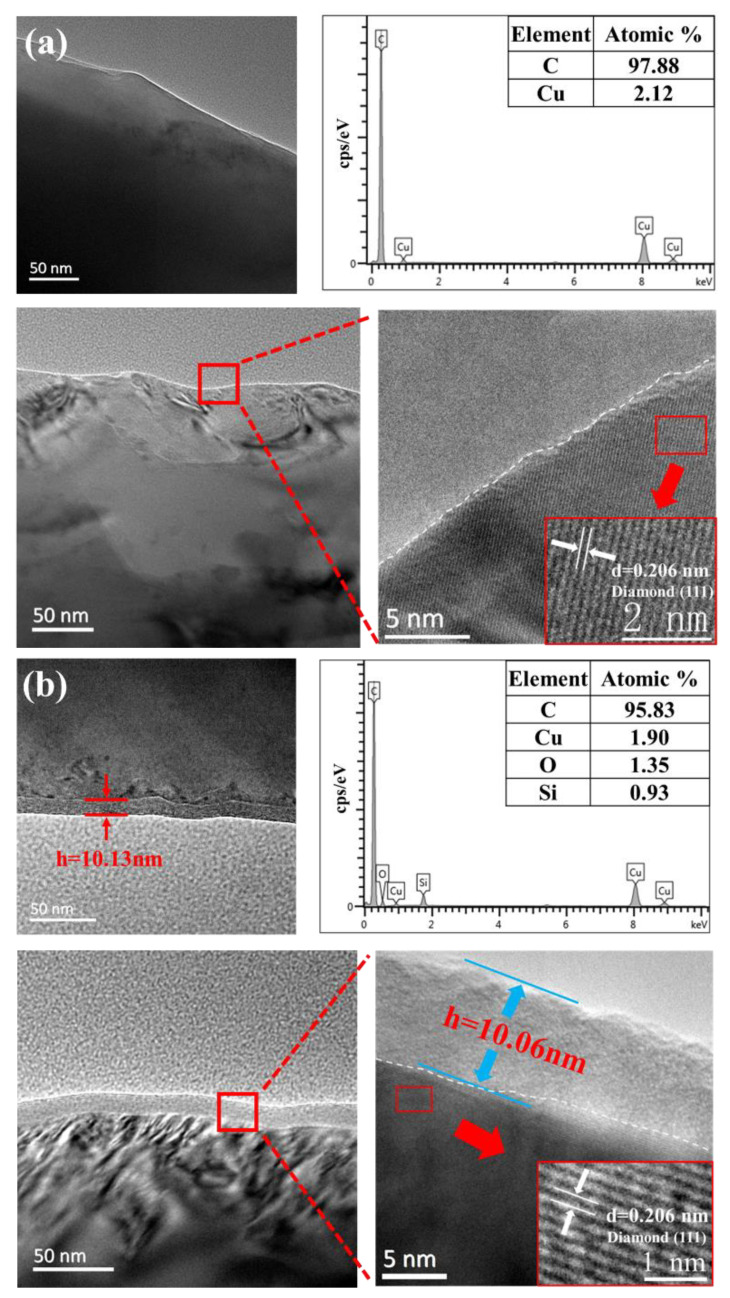
TEM images and EDS spectrum of (**a**) pure diamond and (**b**) diamond/SiO_2_ abrasive particles.

To give a better understanding the microstructure and elemental composition of diamond/SiO_2_ composite abrasives, TEM images and an EDS spectrum of pure diamond and diamond/SiO_2_ composite abrasives are demonstrated in Figure 3. The TEM images and EDS spectrum exhibited in Figure 3a clearly show that the surface of pure diamond is smooth and not covered with other impurities. As presented in the HRTEM image, the lattice fringes of the detected area are clearly visible, which reveals that the pure diamond is crystalline and that the lattice fringe spacing is 0.206 nm, corresponding to the (111) crystal plane. From the EDS elemental map of pure diamond, C and Cu elements could be found in the detection area. Among them, part of C element comes from diamond, the other part stems from carbon film, and Cu element is attributed to copper mesh. From the above results, the pure diamond has high purity and no impurities on the surface, which is conducive to the coating process of diamond abrasive.

Figure 3b exhibits the microstructure and elemental composition of diamond/SiO_2_ composite abrasives. It is noticed that there is an obvious boundary between the core and shell [24]; under the HRTEM, the lattice fringes can be observed in the core; and the spacing of lattice fringes is 0.206 nm, which corresponds to the pure diamond in Figure 3a, whereas the fact that the shell has no lattice fringes confirms the amorphous structure of coating layer. Furthermore, the coating layer is uniform and dense, with a thickness of 10 nm. The EDS spectrum of diamond/SiO_2_ composite abrasives is illustrated in Figure 3b. Compared with pure diamond, besides C and Cu elements, Si and O elements could be found in the composite abrasives. Combined with the TEM images, it can be explained that the coating layer is amorphous SiO_2_.

**Figure 4:**
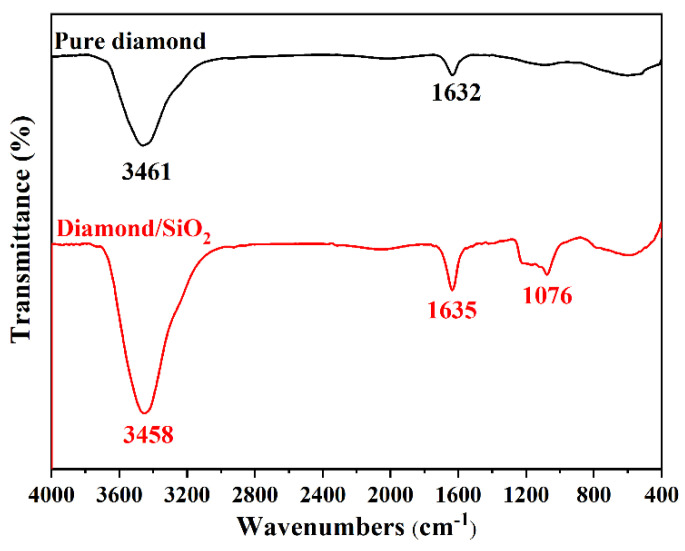
FT-IR spectra of pure diamond and diamond/SiO_2_ composite abrasives.

An attempt can be made to research the surface functional groups of abrasives by using FTIR spectroscopy. Figure 4 presents the FT-IR spectra of pure diamond and diamond/SiO_2_ composite abrasives. For pure diamond, the absorption peaks around 3461 cm^−1^ and 1632 cm^−1^ are attributed to O-H stretching and bending vibrations of adsorbed water [25], respectively. In contrast, the peak at 1076 cm^−1^ appears in the spectra of diamond/SiO_2_ composite abrasives, which is ascribed to the asymmetric stretch vibration of Si-O-Si [26], indicating that SiO_2_ is grafted on the surface of the diamond.

**Figure 5:**
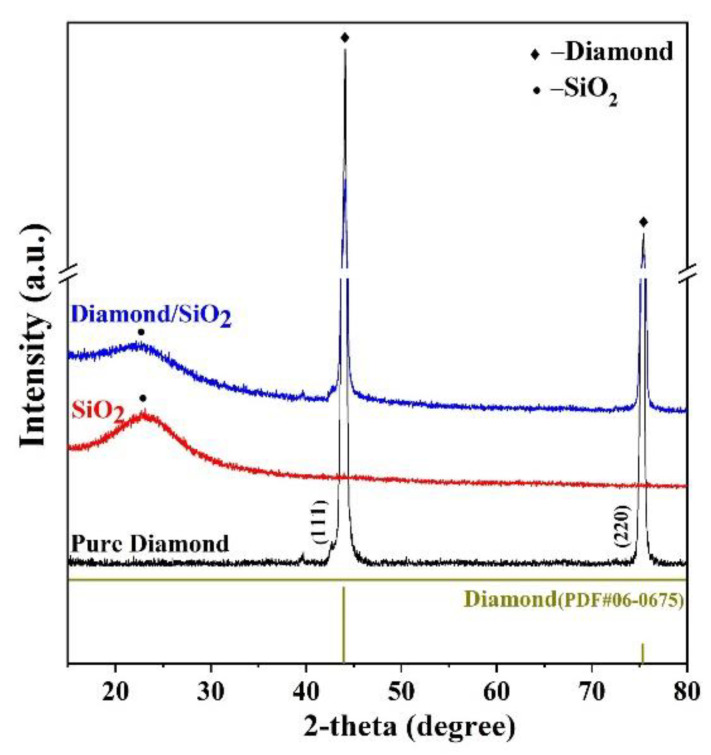
XRD patterns of pure diamond, pure SiO_2_, and diamond/SiO_2_ composite abrasives.

The XRD patterns of pure diamond, amorphous SiO_2_, and diamond/SiO_2_ composite abrasives are demonstrated in Figure 5. As we can see, the characteristic diffraction peaks at 2-theta = 43.9° and 75.3° correspond to the (111) and (220) lattice planes [20], respectively, which could be indexed as the diamond standard card (PDF#06-0675). The diffraction peak of amorphous SiO_2_ is a wide and low diffusion peak near 24.3°. The feature peaks of diamond/SiO_2_ composite abrasives match well with all the diffraction peaks, suggesting that the composite abrasives both contain crystalline diamond and amorphous SiO_2_, and they have a stable interfacial bonding in core-shell structure, which corresponds to the TEM characterization results. From the above characterization results, it can be seen that diamond/SiO_2_ core-shell structure composite abrasives were successfully synthesized and that amorphous SiO_2_ with a thickness of about 10 nm was closely coated on the diamond surface.

### 3.2. Polishing Test

Under the same testing conditions, sapphire wafers were polished with pure diamond and diamond/SiO_2_ composite abrasives to investigate the polishing performance of as-prepared diamond/SiO_2_ composite abrasives.

**Figure 6:**
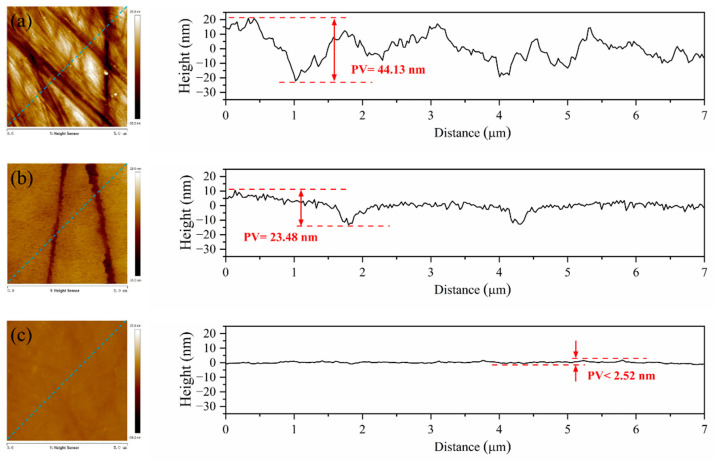
Morphologies and the corresponding profile curves of (**a**) the pristine sapphire wafer and sapphire wafers polished by (**b**) pure diamond and (**c**) diamond/SiO_2_ composite abrasives.

Figure 6 illustrates surface morphology and corresponding profile curves of the sapphire wafers before and after polishing with pure diamond and diamond/SiO_2_ composite abrasives, respectively. As shown in Figure 6a, the AFM images of pristine sapphire wafers demonstrate poor flatness, rough surface, and numerous scratches, with a maximum scratch depth of more than 22.3 nm, as well as the PV (peak-to-valley) value of 44.13 nm. Figure 6b presents the morphology of the sapphire wafer after polishing with pure diamond. On the whole, the surface flatness has been improved. However, two obvious scratches pass through the sapphire surface, with a depth of 13.1 nm, and the value of PV is 23.48 nm. It could be found that pure diamond abrasives will inevitably bring pits and scratches, which offer limited improvement to the surface quality of sapphire. The morphology of a sapphire wafer after polishing it with diamond/SiO_2_ composite abrasives is exhibited in Figure 6c, in which all the obvious defects, such as bumps and scratches on the pristine surface, are eliminated. By contrast, the micro profile curve is smooth, and the PV value is the lowest, at 2.52 nm. It can be concluded that the surface morphology of sapphire wafers polished by diamond/SiO_2_ composite abrasives is superior to that treated by pure diamond, resulting in a smoother and scratch-free surface.

**Figure 7:**
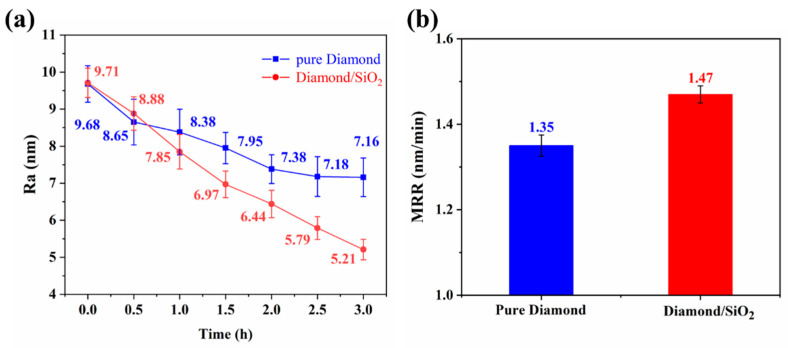
(**a**) Surface roughness (Ra) and (**b**) MRR of the sapphire wafer polished with pure diamond and diamond/SiO_2_ composite abrasives.

The roughness at different processing time periods and the MRR of a sapphire wafer polished by pure diamond and diamond/SiO_2_ composite abrasives are exhibited in Figure 7, in which the roughness shows a downward trend in 3 h of processing with both abrasives under the same parameters. Because of the limitations of pure diamond abrasives, the roughness finally tends to be flat, whereas the roughness of composite abrasives always keeps significantly dropping. Finally, the Ra value of pure diamond decreased to 7.16 nm, and the composite abrasives reached 5.21 nm, which decreased by 27.2%. Meanwhile, after machining a sapphire wafer with diamond/SiO_2_ composite abrasives, the MRR is 1.47 nm/min, which is 8.8% larger than the 1.35 nm/min of pure diamond. Along with the morphology of sapphire wafers shown in Figure 6, it can be concluded that compared with pure diamond, the diamond/SiO_2_ composite abrasives have achieved more-significant polishing performance on sapphire wafers with lower surface roughness and a higher MRR.

### 3.3. Polishing Mechanism

The mechanical-chemical polishing of sapphire wafers includes chemical corrosion and mechanical action, which complement and promote each other. On the basis of these two aspects, the mechanism of improving the polishing quality of diamond/SiO_2_ composite abrasives was investigated. 

**Figure 8:**
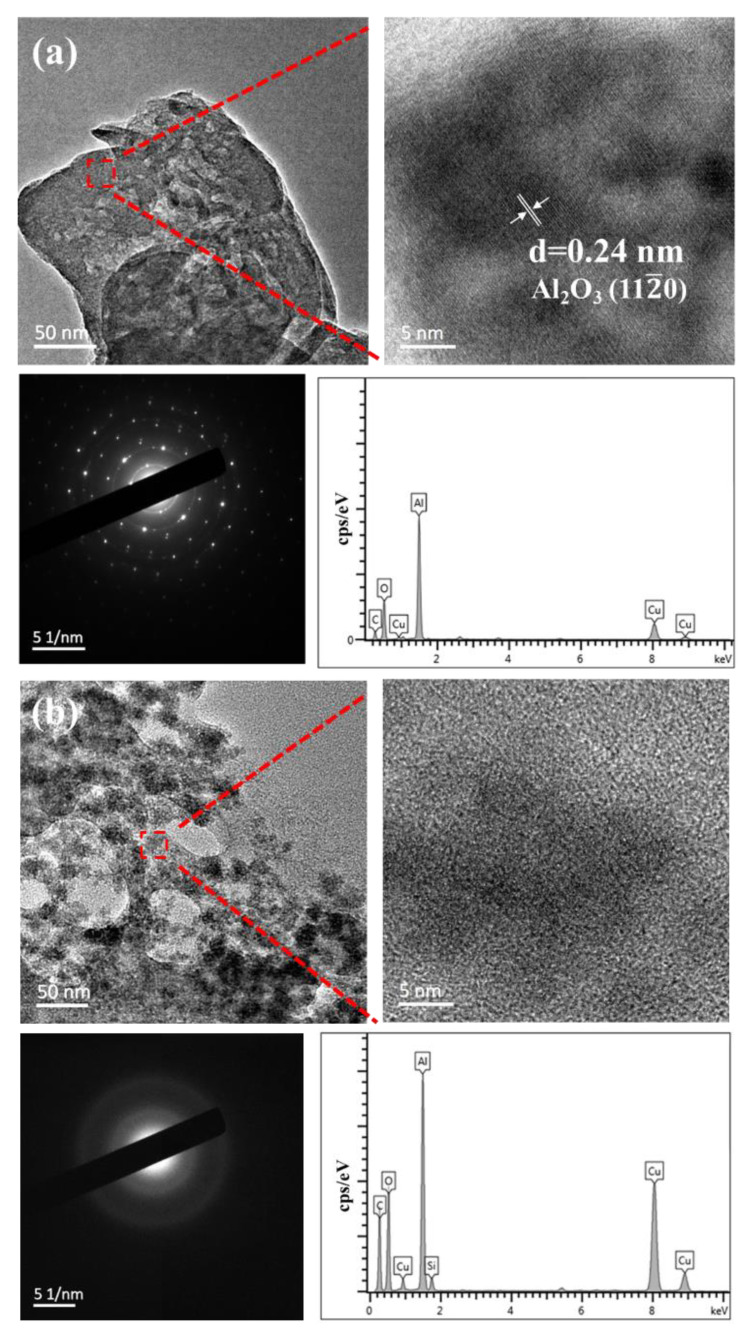
TEM images, HRTEM images, SAED patterns, and EDS spectrum of wear debris produced by (**a**) pure diamond, (**b**) diamond/SiO_2_ composite abrasives.

The wear debris, which was removed from the surface of sapphire wafers during polishing, can be used to analyze the polishing mechanism. TEM images, HRTEM images, EDS spectra, and SAED patterns of the wear debris generated by pure diamond and composite abrasives during polishing are presented in Figure 8. The C and Cu elements in the EDS spectra come from the copper mesh plated with carbon support film. As shown in Figure 8a, the wear debris produced by pure diamond is in the form of a block with a size of about 200 nm [27]. Under HRTEM, lattice stripes can be observed with a spacing of 0.24 nm, which matches the (11$\bar{2}$0) crystal plane of sapphire. Combined with the EDS energy spectrum, it can be preliminarily determined as sapphire wear debris. The corresponding SAED pattern shows regular diffraction points, but polycrystalline concentric rings can also be vaguely observed, demonstrating that the wear debris was most likely both crystalline and amorphous, indicating that the pure diamond abrasives are removed mainly by a single mechanical method in the polishing process. Figure 8b illustrates the wear debris generated by diamond/SiO_2_ composite abrasives, and that debris is in the shape of fragments, showing the size of dozens of nanometers. Under the detection area shown in the figure, the HRTEM image has no lattice fringes, and the SEAD pattern presents a large diffuse halo, indicating that the wear debris is amorphous. Furthermore, the EDS spectrum contains not only Al and O elements but also an Si element. It can be inferred that the SiO_2_ shell reacts with sapphire in the process of polishing to form a new amorphous Al_2_O_3_-SiO_2_ compound, which means that the material removal process combines chemical corrosion and mechanical action [28].

**Figure 9:**
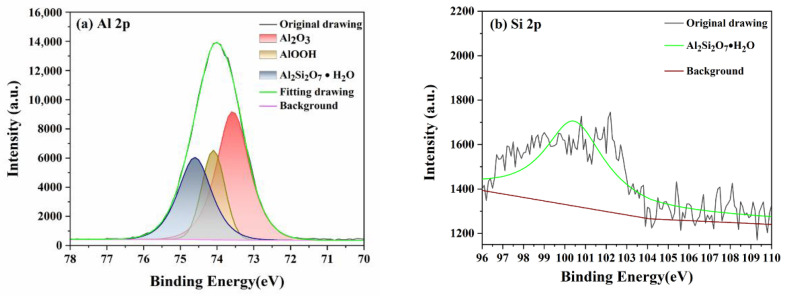
(**a**) Al 2p and (**b**) Si 2p narrow scan spectra on sapphire surface polished by diamond/SiO_2_ composite abrasives.

XPS was used to characterize the composition and existing form of elements in the sample, which can confirm the chemical corrosion mechanism in the polishing process. Figure 9 demonstrates the XPS spectra of the Al and Si elements on the sapphire wafer surface polished by diamond/SiO_2_ composite abrasives. According to the narrow scanning spectrum of Al 2p shown in Figure 9a, there are three main chemical states of Al on the surface of sapphire. The peak, centered at 73.57 eV, corresponds to Al_2_O_3_, which is the main component of sapphire wafer, and peaks at 74.10 eV and 74.60 eV can be assigned to AlOOH and Al_2_Si_2_O_7_⋅H_2_O [29], respectively. From Figure 9b, the peak of Si 2p with the binding energy of 100.43 eV could be attributed to Al_2_Si_2_O_7_⋅H_2_O [30]. This observation indicates that the SiO_2_ shell of composite abrasives underwent a solid-state chemical reaction with the surface of sapphire, which yields AlOOH with a Mohs hardness of 3–3.5 and Al_2_Si_2_O_7_⋅H_2_O with a Mohs hardness of 4. Possible reactions can be summarized as follows:

(5) $${\mathrm{Al}}_{2}O_{3}+H_{2}O\to 2\mathrm{AlOOH}\;$$

(6) $$2\mathrm{AlOOH}+2{\mathrm{SiO}}_{2}\to {\mathrm{Al}}_{2}{\mathrm{Si}}_{2}O_{7}\cdot H_{2}O\;$$

**Figure 10:**
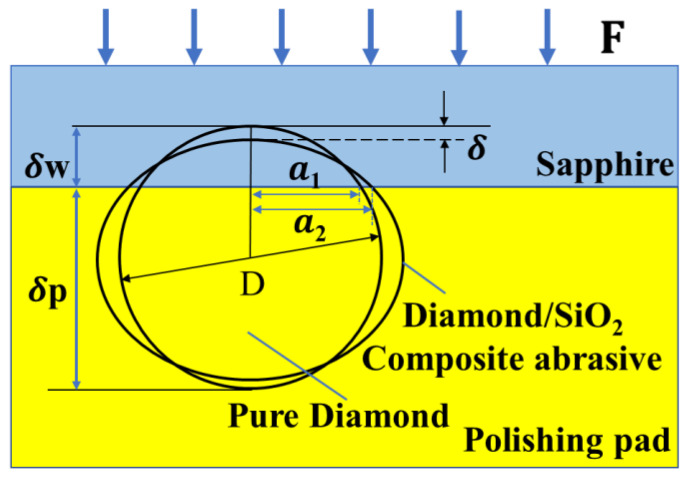
Microcontact diagram of pure diamond and diamond/SiO_2_ composite abrasive during the polishing process.

In addition, the types and properties of the abrasive particles are the keys to mechanical action, which affects the whole mechanical-chemical polishing process. Compared with pure diamond, the composite abrasives coated with amorphous SiO_2_ shells have a lower elastic modulus. On the basis of the microcontact mechanics model proposed by Chen [31], and given the deformation of abrasive particles in the machining process, the microcontact model between sapphire wafer, abrasive particles, and polishing pads was established, as shown in Figure 10. Equations (7)–(9) demonstrated the calculation formulas for the indentation depth into the sapphire wafer and the deformation of abrasive particles. Under the action of polishing pressure F, the indentation depth $\delta_{w}$ pressed into the sapphire wafer is reduced because of the lower Young’s modulus $E_{s}$ of composite abrasives. As a result, the diamond/SiO_2_ composite abrasives became an ellipsoid during the polishing process, which makes the contact stress lower and more uniform. The reduction of the indentation depth can effectively avoid deep scratches and serious damage, which has a decisive impact on improving the surface roughness. In this case, the roughness of a processed sapphire wafer will be improved when diamond/SiO_2_ composite abrasives are used in polishing, but the MRR will decrease because of the reduction in the indentation depth, which results in a weaker plow effect.

(7) $$\delta ={\left(\frac{9F^{2}}{8DE_{sw}^{2}}\right)}^{\frac{1}{3}}$$

(8) $$\frac{1}{E_{sw}}=\frac{1-v_{s}{}^{2}}{E_{s}}+\frac{1-v_{w}{}^{2}}{E_{w}}$$

(9) $$\delta_{w}=D-\delta -\delta_{p}=D-\delta \left[\;1+{\left(\frac{E_{sw}}{E_{sp}}\right)}^{\frac{3}{2}}\right]$$

where F is the polishing pressure, δ is the deformation of the particle; D is the diameter of the particle; $E_{sw}$ is the Young’s modulus of the particle and wafer pair; $E_{s}$ and $v_{s}$ are the Young’s modulus and the Poisson’s ratio of the abrasive particle, respectively; $E_{w}$ and $v_{w}$ are the Young’s modulus and the Poisson’s ratio of the wafer, respectively; $E_{sp}$ is the Young’s modulus of the particle and pad pair; $\delta_{p}$ is the indentation depth of the particle into the polishing pad; and $\delta_{w}$ is the indentation depth of the particle into the sapphire wafer [31].

**Figure 11:**
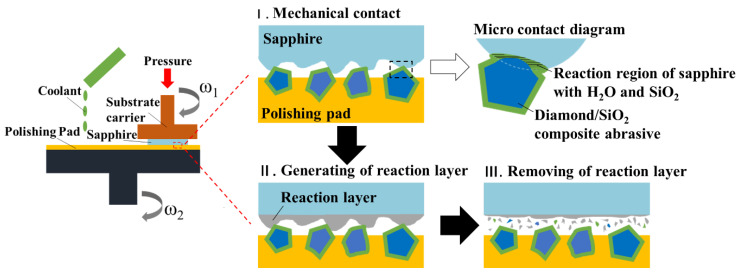
Material removal model of diamond/SiO_2_ composite abrasives for polishing sapphire wafers.

In fact, when the diamond/SiO_2_ composite abrasives are used in the polishing process of sapphire, the larger elastic deformation also obtains a larger contact area with the sapphire wafer, such that the solid-state reaction can proceed more continuously and fully [32]. Subsequently, the softening reaction products can be easily removed by the mechanical action of abrasives, which is more significant than the plow effect, thus further improving the MRR. Therefore, only by relying on the chemical corrosion and mechanical action of composite abrasives to balance and promote each other is it possible to at the same time improve the surface roughness and the MRR. Figure 11 shows the material removal model of diamond/SiO_2_ composite abrasives for polishing sapphire wafers.

## 4. Conclusions

By depositing SiO_2_ on diamond surface, core-shell structured composite abrasives can be synthesized using a simplified sol-gel method. The diamond/SiO_2_ core-shell composite abrasives showed excellent polishing performance in green polishing technology without corrosive slurry. Compared with pure diamond, composite abrasives obtained ultrasmooth and low-damage sapphire surfaces, which effectively reduced the surface roughness by 27.2%, accompanied by an 8.8% improvement in the MRR. The reduction in surface roughness may have been caused by the lower Young’s modulus of diamond/SiO_2_ composite abrasives, which resulted in the decrease in indentation depth pressed into the sapphire wafer. In addition, chemical reactions occurred between the sapphire and SiO_2_ shell during the polishing process, which yielded AlOOH and Al_2_Si_2_O_7_ with low hardness. Meanwhile, the sufficiently continuous solid-state reaction brought by soft SiO_2_ shells enhanced the MRR in cooperation with the mechanical removal action of composite abrasives. Thus, diamond/SiO_2_ composite abrasives are well-defined abrasives that meet the practical requirements of the high surface quality and high MRR of sapphire wafers.
